# Structural Biology-Guided Multiomics of the Human Microbiome

**DOI:** 10.1063/4.0000935

**Published:** 2025-10-27

**Authors:** Mattthew R. Redinbo

**Affiliations:** University of North Carolina, Chapel Hill, NC, USA

## Abstract

The microbiome is a vast repository of biological active factors that directly influence human physiology, transitions to disease, and therapeutic efficacy. While DNA sequencing has revolutionized our understanding of the differential complexity of the gut microbiome in health and disease, it fails to be able to pinpoint the specific proteins that are driving catalytic outcomes in a complex human fecal sample. Here we show that the distinct activities of diverse gut microbial enzymes on a range of distinct substrates can be defined using structural-biology guided mining of whole genome sequencing data. Furthermore, we couple these data with activity-based probe-enabled proteomics collected from ex vivo human samples from healthy individuals and patients with disease. Together, this approach can define the gut microbial enzymes that drive the reactivation of the cancer, pain and immunosuppressant drugs. We also demonstrated that specific forms of gut microbial enzymes drive changes in both the intestinal and systemic levels of serotonin, and that extant drugs have an unexpected and significant impact on endobiotic levels by inhibiting these microbial factors. These approaches show that the inclusion of structural biology in mining metagenomics and proteomics data critically guides the analyse of multiomics results to define the effects that non-host enzymes have on host physiology and disease. Utilizing structural biology results in this way may provide a new chapter to the impact that structural chemistry will have on biological discovery.

